# Changes in diversity and community assembly of jumping spiders (Araneae: Salticidae) after rainforest conversion to rubber and oil palm plantations

**DOI:** 10.7717/peerj.11012

**Published:** 2021-03-04

**Authors:** André Junggebauer, Tamara R. Hartke, Daniel Ramos, Ina Schaefer, Damayanti Buchori, Purnama Hidayat, Stefan Scheu, Jochen Drescher

**Affiliations:** 1 Department of Animal Ecology, J-F. Blumenbach Institute for Zoology and Anthropology, University of Göttingen, Göttingen, Germany; 2 Department of Plant Protection, Faculty of Agriculture, Bogor Agricultural University, Bogor, Indonesia; 3 Center for Transdisciplinary and Sustainability Sciences, IPB University, Bogor, Indonesia; 4 Centre of Biodiversity and Sustainable Land Use, University of Göttingen, Göttingen, Germany

**Keywords:** Deforestation, Rainforest, Oil palm, Land-use change, Rubber, Cash Crops, Spiders, South-East Asia, Diversity

## Abstract

Rainforest conversion into monoculture plantations results in species loss and community shifts across animal taxa. The effect of such conversion on the role of ecophysiological properties influencing communities, and conversion effects on phylogenetic diversity and community assembly mechanisms, however, are rarely studied in the same context. Here, we compare salticid spider (Araneae: Salticidae) communities between canopies of lowland rainforest, rubber agroforest (“jungle rubber”) and monoculture plantations of rubber or oil palm, sampled in a replicated plot design in Jambi Province, Sumatra, Indonesia. Overall, we collected 912 salticid spider individuals and sorted them to 70 morphospecies from 21 genera. Salticid richness was highest in jungle rubber, followed by rainforest, oil palm and rubber, but abundance of salticids did not differ between land-use systems. Community composition was similar in jungle rubber and rainforest but different from oil palm and rubber, which in turn were different from each other. The four investigated land-use systems differed in aboveground plant biomass, canopy openness and land use intensity, which explained 12% of the observed variation in canopy salticid communities. Phylogenetic diversity based on ~850 bp 28S rDNA fragments showed similar patterns as richness, that is, highest in jungle rubber, intermediate in rainforest, and lowest in the two monoculture plantations. Additionally, we found evidence for phylogenetic clustering of salticids in oil palm, suggesting that habitat filtering is an important factor shaping salticid spider communities in monoculture plantations. Overall, our study offers a comprehensive insight into the mechanisms shaping communities of arthropod top predators in canopies of tropical forest ecosystems and plantations, combining community ecology, environmental variables and phylogenetics across a land-use gradient in tropical Asia.

## Introduction

Tropical rainforests are exceptionally species rich, contain much of the world’s biodiversity and are one of the biggest carbon sinks in the world (Lowman & Nadkarni, 1995; Sodhi et al., 2004; Soepadmo, 1993). Rainforest conversion is one of the main reasons for worldwide biodiversity loss (Brooks et al., 2002; Pimm & Raven, 2000; Sala, 2000), and in Southeast Asia, deforestation rates of natural habitats such as lowland rainforests are among the highest of all tropical regions (Achard et al., 2014; Hansen et al., 2013; Sodhi et al., 2004; Sala, 2000). Since the 1950s, commercial timber extraction as well as cultivation of rubber (*Hevea brasiliensis*) and oil palm (*Elaeis guineensis*) continue to be the main drivers of deforestation in Southeast Asia (Austin et al., 2017; Laumonier et al., 2010). By 2010, about 70% of the original lowland rainforest of Sundaland, comprising the Malay Peninsula, Borneo, Sumatra and Java, were lost to deforestation (Wilcove et al., 2013). Ongoing rainforest conversion could result in the loss of up to 42% of the region’s biodiversity by 2100 (Sodhi et al., 2004).

Arthropods constitute the majority of animal biomass and biodiversity in tropical ecosystems (Fittkau & Klinge, 1973; Samways, 2005), and arguably most arthropods colonize tree canopies (Hamilton et al., 2010). Arboreal arthropods are thus a crucial part of global diversity (Floren et al., 2011) and contribute significantly to overall ecosystem functioning as herbivores, predators, parasites and parasitoids, seed dispersers and pollinators (Samways, 2005; Schowalter, 2011). Spiders (Araneae) are one of the most abundant generalist predator groups in rainforests (Benítez-Malvido, Martínez-Falcón & Durán-Barrón, 2020), agroecosystems (Birkhofer, Entling & Lubin, 2013) and other terrestrial ecosystems (Nyffeler & Birkhofer, 2017). As high-level predators in arthropod food chains, they are crucial for natural pest control and ecosystem functions (Birkhofer et al., 2008; Birkhofer, Entling & Lubin, 2013; Lefebvre et al., 2017; Schmitz, 2007). Their predatory function and efficacy in pest control is particularly high in the tropics (Michalko et al., 2019). However, rainforest conversion may threaten these services, as re-colonization of disturbed areas likely depends on natural rainforest as a source of high spider richness (Benítez-Malvido, Martínez-Falcón & Durán-Barrón, 2020; Floren & Deeleman-Reinhold, 2005). Recently, it has been demonstrated that the complexity of ground-spider communities decreases following rainforest conversion to rubber and oil palm plantations (Potapov et al., 2020). Similarly, among the limited literature available on this topic, Floren & Deeleman-Reinhold (2005), Floren et al. (2011), Zheng, Li & Yang (2015) and Zheng et al. (2017) found less complex spider communities in degraded forest or tree plantations than in undisturbed forests in Sabah, Malaysia and Xishuangbanna Dai, China. Overall, however, the effects of tropical rainforest conversion to cash-crop monocultures on spiders in tree and palm canopies have been little studied.

In addition, the mechanisms governing community assembly of spiders are little understood. We are aware of only two studies on this topic. They found that adaptive radiation and harsh environmental conditions shaped the assembly of spider communities in Hawaii (Gillespie, 2004) and the Iberian Peninsula (Cardoso, 2012). Despite the prevalence of spiders in tropical canopies, we are unaware of any studies that have quantified the mechanisms influencing canopy-spider community assembly after rainforest conversion into cash-crop monoculture plantations. However, it has been demonstrated that tree canopy openness and vegetation complexity, for example, coverage by herbs, shrubs and epiphytes, affect local spider communities (Méndez-Castro et al., 2020; Méndez-Castro et al., 2018; Stenchly et al., 2011; Zheng, Li & Yang, 2015).

The structure of communities and their changes in space and time are often analysed using diversity metrics (Dopheide et al., 2020; Santini et al., 2017). Species richness, a common measure of diversity and an important aspect of community composition, represents a snapshot limited to one point in time. Combined with the branch lengths in a phylogenetic tree (Faith, 1992), the resulting Phylogenetic Diversity (PD), Net Relatedness Index (NRI) and Nearest Taxon Index (NTI) provide insight into the evolutionary history of species assemblages by incorporating inferred phenotypic variation between species (Cadotte, Cardinale & Oakley, 2008; Miller et al., 2018). The general assumption that ecological traits show phylogenetic signal, that is, closely related species are ecologically more similar than distantly related species, allows inferences on competitive exclusion or habitat filtering as the main factors in community assembly, based on the phylogenetic relationships within a community (Vamosi et al., 2009; Webb et al., 2002). Communities structured predominantly by competitive exclusion consist of more distantly related species than would be expected by chance, as closely related species which share limited resources cannot coexist, resulting in overdispersal in the community phylogenetic tree (phylogenetic overdispersion). By contrast, communities influenced by habitat filtering are associated with phylogenetic and phenotypic clustering: the environment functions as a filter that selects for species with similar traits, which tend to be more closely related than random assemblies (Webb et al., 2002).

Here, we combined a community ecological approach with DNA sequence data to understand how canopy spider communities change after rainforest conversion to cash crop monoculture plantations, and which factors drive those changes. We investigated salticid spiders (Araneae: Salticidae), the most speciose spider family worldwide, with a total of 6,216 described species (World Spider Catalog, 2020). Salticids are by far the most diverse family in Indonesia and New Guinea, with 657 described species, followed by Araneidae (244 species; Ramos, 2020). They are one of the dominant spider families in rainforest canopies (Floren & Deeleman-Reinhold, 2005; Zheng, Li & Yang, 2015) and we took them to represent cursorial spiders in these habitats. Specifically, we compared abundance, richness and community composition of salticid spider communities from lowland rainforest, rubber agroforest (“jungle rubber”; Rembold et al., 2017; Gouyon, De Foresta & Levang, 1993), and smallholder monoculture plantations of rubber or oil palm. We also investigated the role of selected environmental factors on canopy salticid community composition in the four land-use systems. Further, we sequenced the 28S rDNA and *COI* to compare phylogenetic diversity of salticid spiders among the studied land-use systems, and then used the Net Relatedness Index and Nearest Taxon Index (Webb et al., 2002) to investigate the relative influence of habitat filtering and competition on salticid community assembly in all four land-use systems.

Previous findings from the same study sites indicated reduced diversity and abundance of a range of arthropod taxa with the conversion of lowland rainforest into monoculture plantations (Barnes et al., 2014; Clough et al., 2016; Nazarreta et al., 2020; Potapov et al., 2020). Thus, we hypothesized that (1) abundance and richness of canopy salticids decrease continuously across the land-use gradient, with highest values in lowland rainforest, intermediate in jungle rubber and lowest in monocultures of rubber or oil palm. Further, we hypothesized (2) that community composition of salticid spiders differs between the land-use systems, allowing us to explore the role of environmental factors on community assembly. Lastly, we hypothesized that (3) rainforest conversion strengthens the effect of habitat filtering, which would be reflected in lower phylogenetic diversity and phylogenetic clustering in the community assembly of canopy salticids in oil palm and rubber plantations.

## Materials and Methods

### Sampling and identification

Salticid spiders were collected in the dry season 2017 (June–August) from the research plots of the EFForTS project in Jambi Province, Sumatra, Indonesia (Collection permit: S.710/KKH-2/2013, PHKA Jakarta, Indonesia; Research permit: 131/SIP/FRP/E5/Dit.KI/V/2017, RISTEKDIKTI Jakarta, Indonesia). Access to field sites (plots) was granted through more than 35 bilateral agreements of Jambi University (UNJA, EFForTS partner University) with PT REKI (Perseroan Terbatas Restorasi Ekosistem Indonesia), which manages Harapan Rainforest, the Bukit Duabelas National Park administration, as well as individual smallholder farmers of rubber, jungle rubber and oil palm from Jambi Province. Jambi Province has a tropical humid climate with two peak rainy seasons in March and December, mean monthly rainfall above 100 mm and an average monthly temperature of 26.7 °C throughout the year (Drescher et al., 2016; Meijide et al., 2018). In a nested, replicated plot design, eight 50 × 50 m research plots were established in each of four land-use systems: (A) primary degraded rainforest (selectively logged primary forest; for terminology see Margono et al., 2014), (B) jungle rubber (extensive rubber agroforest system with rubber trees planted into secondary or disturbed rainforest, Rembold et al., 2017), and monoculture plantations of (C) rubber (*Hevea brasiliensis)* or (D) oil palm (*Elaeis guineensis*) (Fig. S1; for details see Drescher et al., 2016). The resulting 32 research plots are evenly distributed between two clusters within and adjacent to the forest reserves (a) Bukit Duabelas National Park and (b) Harapan Rainforest. The clusters are about 70 km apart, and henceforth referred to as the “Bukit Duabelas” and “Harapan” landscapes. The landscapes differ with respect to soil structure: the Acrisols in Bukit Duabelas are clay-dominated, while Harapan Acrisols have a higher loam content (Allen et al., 2015; Guillaume et al., 2018). Canopy salticids were sampled from three target canopies per plot via canopy fogging using 50 ml DECIS 25 EC^®^ insecticide (BayerCropScience, active ingredient delthamethrine, 25 g/L) dissolved in 4 L petroleum white oil. Stunned or dead spiders were collected after a 2 h drop time in eight 1 × 1 m traps beneath each target canopy (described in more detail in Nazarreta et al., 2020), then cleaned and stored in 96% EtOH. Spiders were removed from the arthropod mix and determined to morphospecies; the resulting species abundance data was pooled for each plot.

From a total amount of 912 salticid spiders (Araneae: Salticidae), 677 individuals were identified to morphospecies; 235 juveniles, many newly hatched, were excluded from the analysis as morphological characters for determination were not developed. Morphological identification was supported by images and character guides in Ramos et al. (2019) and Ecotaxonomy.org.

### Statistical analysis

Statistical analyses were conducted using R v3.6.2 (R Core Team, 2019) and visualized with *ggplot2* (Wickham, 2016). Venn Diagrams were produced using a customized script based on (limma::VennCounts, Ritchie et al., 2015). For each land-use type, we generated rank abundance curves (Whittaker, 1965; implemented in RankAbund; Hartke, 2020) to display relative species abundances and species accumulation curves (vegan::specaccum, method = random; Gotelli & Colwell, 2001) to estimate sampling completeness. Abundance (individuals/m^2^, sum of three replicate canopies per plot divided by the total number of traps) had a log normal distribution, and was analysed using a generalised linear model (glm) with family Gaussian and the log link function, while species richness (S; number of species per plot; vegan::specnumber, Oksanen et al., 2018) fit the assumption for linear models (lm). Both initial models contained land use (four levels: rainforest, jungle rubber, rubber, oil palm), landscape (two levels: Bukit Duabelas and Harapan), and their interaction as fixed factors. We included landscape in the models as a fixed factor because different soil textures may indirectly influence canopy arthropods by changing plant characteristics. We used step-wise simplification to arrive at the final model with the lowest AIC. Final models were subjected to one-way analysis of variance (ANOVA; Chambers & Hastie, 1992). Multiple comparison between factor levels of dependent variables contributing significantly to the model (*p* < 0.05) were performed using Tukey’s HSD (multcomp::glht, Hothorn, Bretz & Westfall, 2008) with Holm’s adjustment (Holm, 1979).

Community composition was analysed using non-metric multidimensional scaling (NMDS; Kruskal, 1964; vegan::mds; Bray Curtis dissimilarity index; *k* = 5, stress = 0.098). Multivariate analysis of variance (MANOVA; Hand & Taylor, 1987) was used to test whether land use or landscape explain the species patterns in the NMDS. We also performed a model-based analysis of multivariate data (mvabund::many.glm; Wang et al., 2012), as this method can outperform distance-based approaches in recovering the true relationships between sites (Hui et al., 2015). Residual versus fitted plots (mvabund::plot.manyglm) indicated that a negative binomial distribution fit best to our multivariate species data. This was followed by a Bayesian ordination (boral::boral; Hui, 2016) for a model-based approach to unconstrained ordination, fitting a pure latent variable model (LVM). The model used a negative binomial distribution, a site-level random effect to focus on community composition rather than abundance, and two latent variables to create a biplot analogous to NMDS plots (Hui et al., 2015), which was visualised using *ggboral* (Bedward, 2020).

Canonical correspondence analysis (CCA; Ter Braak, 1986; Legendre & Legendre, 2012; vegan::cca) was conducted to evaluate the relationship between the distribution of salticid spiders and environmental factors, such as temperature (°C; Meijide et al., 2018), relative humidity (%; Meijide et al., 2018), aboveground biomass (AGB, Mg/ha Guillaume et al., 2018), stand structural complexity (SSCI; Zemp et al., 2019a), canopy openness (%; Kotowska et al., 2015) and the land use intensity index (LUI; Brinkmann et al., 2019), which is calculated from the number of planted trees per hectare and the quantities of fertilizers (kg/year) and herbicides (L/year) used. These environmental variables were measured in the same sampling sites where we collected salticid spiders. As indices (AGB, SSCI, LUI) or consequences (temperature, humidity) of habitat structure, they reflect aspects of habitat complexity known to influence spider community assembly (Floren & Deeleman-Reinhold, 2005; Oxbrough et al., 2005; Stenchly et al., 2011; Uetz, 1991; Wise, 1993; Zheng, Li & Yang, 2015; Ziesche & Roth, 2008). Relative humidity was excluded from the analysis as the Pearson correlation coefficient indicated an almost perfect linear relationship (0.94) with mean temperature (Fig. S2). The global model containing all other environmental variables (Pearson correlation coefficients < 0.90) was significant under one-way ANOVA (Chambers & Hastie, 1992), so forward selection was used to rank environmental variables according to their importance (Blanchet, Legendre & Borcard, 2008). Forward selection used the alpha significance level and the adjusted coefficient of multiple determination (R²_a_) (vegan::ordiR2step), calculated in the global model, as stopping criteria (Blanchet, Legendre & Borcard, 2008) and ran for 999 permutations. The variance inflation factors (VIF; Akinwande, Dikko & Samson, 2015) from the final model indicated no redundancies or problematic correlations, as variables with a VIF above 5 were eliminated during data exploration or by forward selection from the global model.

### Molecular analysis

DNA was extracted separately from one hind leg of up to three individuals per morphospecies with the Agencourt DNAdvance Kit (Beckman Coulter, Krefeld, Germany). Legs were individually transferred into 94 µl lysis buffer and manually ruptured with a sterile plastic pestle. Then, 2 µl of 1mg/ml chitinase (Sigma–Aldrich, Taufkirchen, Germany) was added and the tissue solution incubated, shaking for 10 minutes at 37 °C. Next, 5 µl of Proteinase K (20 µg/µl, Genaxxon, Ulm, Germany) was added and samples shaken at 55 °C for 5 h. The lysate was transferred, avoiding any remaining tissue to an AB-1127 plate (ThermoFisher Scientific, Dreieich, Germany) and processed on a Biomek 3000 automated workstation (Beckman Coulter, Krefeld, Germany) using the Agencourt DNAdvance standard protocol and an elution volume of 100 µl. Two gene regions were amplified and sequenced: (1) the nuclear-encoded large subunit ribosomal repeat (28S rDNA) (~850 bp), using the primers 28S “O” and 28S “C” (Hedin & Maddison, 2001), and (2) the ~1,000 bp mitochondrial marker cytochrome oxidase I (*COI*) using the primer combination COI_C1-J-1718 (Simon et al., 1994) and COI_C1-N-2776 (Hedin & Maddison, 2001) or the primer pair LCO1490 and HCO2198 (Folmer et al., 1994). Each 25 µl polymerase chain reaction (PCR) contained 2 µl of template DNA, 12.5 µl of SuperHot PCR mastermix (Genaxxon, Ulm, Germany), 1 µl of magnesium chloride (25 mM) and 1 µl of each primer. PCR cycling conditions included an initial activation step at 95 °C for 15 min, 35 amplification cycles (denaturation at 95 °C for 45 s, annealing at 55 °C for 45 s for the ribosomal repeat (28S rDNA) and 52 °C for 45 s for cytochrome oxidase I (*COI*), elongation at 72 °C for 45 s) and a final elongation step at 72 °C for 60 s. The PCR product was sent for purification and bi-directional Sanger sequencing to Seqlab, Göttingen, Germany.

A total of 131 sequences for *COI* (64 of 70 morphospecies) and 125 sequences (59 of 70 morphospecies) for 28S rDNA (for GenBank accession numbers see Table S1) were checked for quality and ambiguous positions were corrected using the electropherograms in Geneious Prime 2019 (http://www.geneious.com, Kearse et al., 2012). 28S rDNA sequences were aligned using “Clustal Omega” in Geneious Prime 2019 and *COI* sequences were aligned as proteins and nucleotides using ClustalW in BioEdit 7.0.5.3 (Hall, 1999). Automatic Barcode Gap Discovery (ABGD; Pmin = 0.001, Pmax = 0.1 and the K2P model) with alignments of both gene regions was used to verify morphospecies identification based on genetic pairwise distances between individuals (https://bioinfo.mnhn.fr/abi/public/abgd/abgdweb.html, Puillandre et al., 2012), resulting in a set of 55 unique molecular species (Table S2). The results of ABGD reduced the dataset from 64 sequenced morphospecies to 60 *COI* lineages and from 59 sequenced morphospecies to 55 28S rDNA lineages by merging eight morphospecies into four unique molecular units. As *COI* sequences provided no resolved phylogenetic tree, we used the Bayesian tree (MrBayes 3.2.7a; Huelsenbeck & Ronquist, 2001) for 28S rDNA for further analyses (Fig. S3).

Phylogenetic Diversity (Faith, 1992), Nearest Relatedness Index and Nearest Taxon Index (Webb et al., 2002) were evaluated using the package picante (Kembel et al., 2010) in R v.3.6.2 (R Core Team, 2019). Phylogenetic Diversity (PD) was calculated with the function pd. NRI and NTI were calculated as the standardized effect sizes of the mean pairwise phylogenetic distance (MPD) and mean nearest taxon distances (MNTD) using the functions ses.mpd and ses.mntd, respectively, each with 999 runs, 1,000 iterations and the model of “independent swap” (Gotelli, 2000). Both metrics were tested against random assembly for each land-use system using *t*-tests, and compared between land-use systems using one-way ANOVAs followed by Holm’s-corrected Tukey’s HSD tests (multcomp::glht, Hothorn, Bretz & Westfall, 2008). Mean values for NRI and NTI are zero in random assemblies; values significantly above or below zero indicate phylogenetic clustering (suggesting habitat filtering) or overdispersal (indicating competitive exclusion), respectively.

## Results

The sampled 677 canopy salticid spider individuals (excl. juveniles, see Methods) were sorted to 70 morphospecies and 21 genera. More than half of the characterized morphospecies (41 of 70) were found in both landscapes, while 25% of all morphospecies (18 of 70) were found only in Harapan and 15% (11 of 70) exclusively in Bukit Duabelas as singletons (Fig. S4A). The highest number of unique morphospecies were found in jungle rubber and oil palm, with 11 species each. On the other hand, seven morphospecies were exclusively found in forest, two in rubber and six species were present in all land-use systems (Fig. S4B).

**Figure 1:**
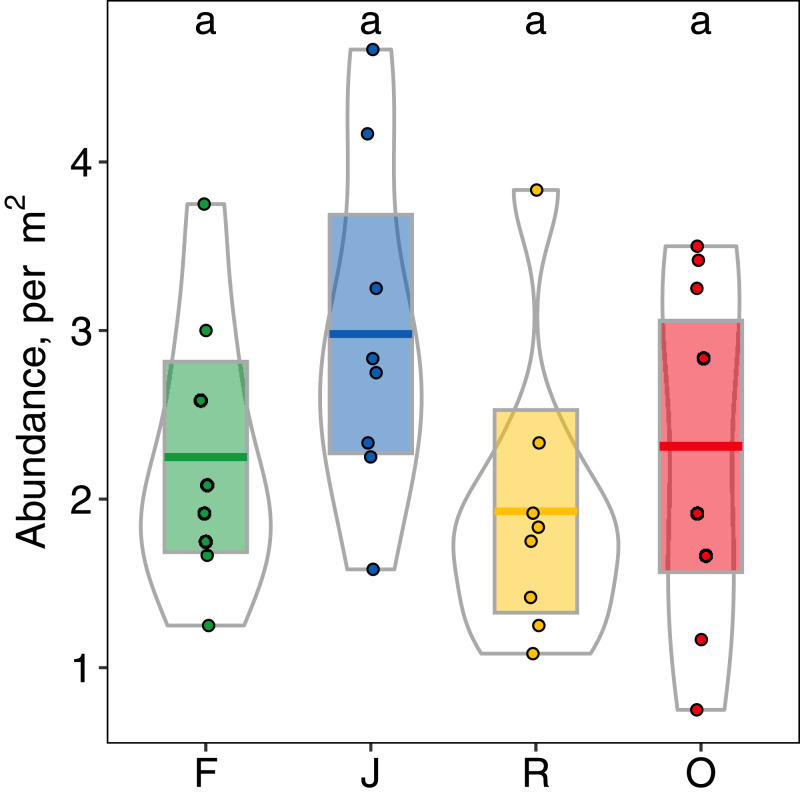
Abundance (individuals/m^2^) of arboreal salticid spider communities across four land-use systems. F = rainforest, J = jungle rubber, R = rubber, O = oil palm. Boxplots show mean (horizontal line), 95% confidence interval (box), density distribution (grey lines) and raw data (dots). Different letters above boxplots mark significant differences as indicated by Holms corrected Tukey HSD, *p* < 0.05.

**Figure 2:**
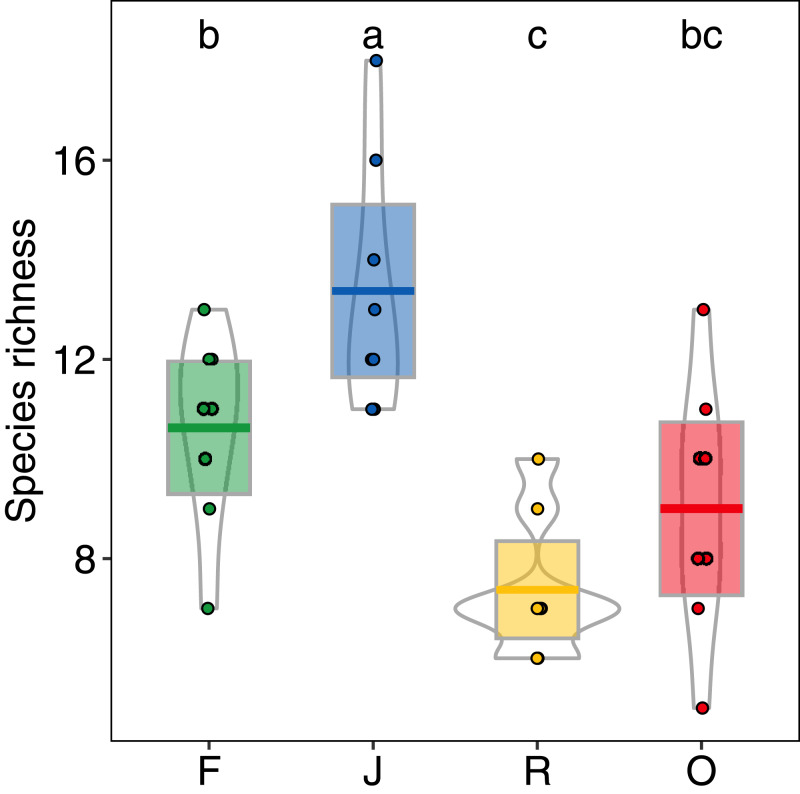
Species richness of arboreal salticid spider communities across four land-use systems. F = rainforest, J = jungle rubber, R = rubber, O = oil palm. Boxplots show mean (horizontal line), 95% confidence interval (box), density distribution (grey lines) and raw data (dots). Different letters above boxplots mark significant differences as indicated by Holms corrected Tukey HSD, *p* < 0.05.

Abundance of salticid spiders did not differ significantly among land-use systems (Fig. 1) or between the two landscapes. Species richness (S), however, was significantly affected by land use (*F*_3,28_ = 11.10, *p* < 0.001) but not landscape (*F*_1,27_ = 0.059, *p* = 0.808). Species richness was 13.00 ± 2.50, mean ± s.d in jungle rubber, which was significantly higher than in all other land-use systems (Fig. 2). Species richness in rainforest was intermediate (*S*_F_ = 10.62 ± 1.92) and significantly higher than in rubber (*S*_R_ = 7.38 ± 1.41), although neither differed from oil palm (*S*_O_ = 9.00 ± 2.51). Species accumulation curves approached an asymptote in oil palm and rubber plantations, but suggested that salticid communities in rainforest and particularly jungle rubber may not have been sampled completely (Fig. S5).

**Figure 3:**
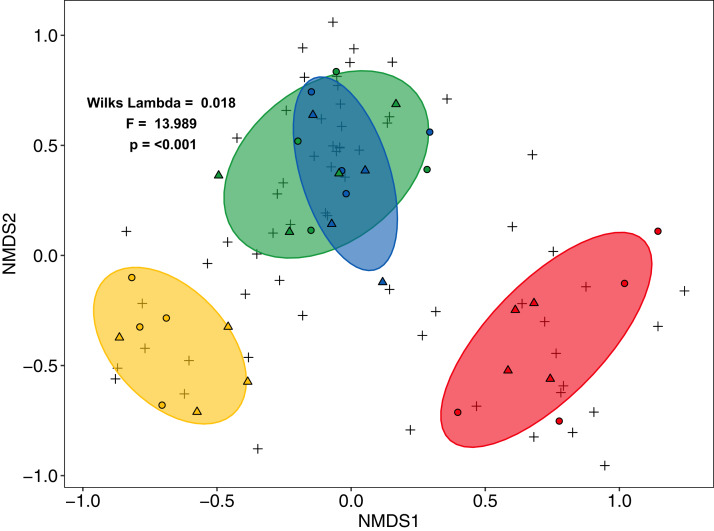
Non-Metric Multi-Dimensional Scaling (NMDS) of arboreal salticid spider communities across four land-use systems. Salticid species (+) in plots (circles = Bukit Duabelas, triangles = Harapan) of rainforest (green), jungle rubber (blue), rubber (yellow) and oil palm (red) based on Bray–Curtis dissimilarity (stress = 0.098, *k* = 5). Ellipses represent 75% confidence intervals.

Community composition of salticid spiders also varied significantly with land use (MANOVA: *F*_3,27_ = 13.98, *p* < 0.001, Wilks Lambda = 0.0180; mvglm: *w*_3,27_ = 12.231, *p* < 0.001), but not landscape (*F*_1,27_ = 2.21, *p* = 0.087, Wilks Lambda = 0.675; *w*_1,27_ = 6.575, *p* = 0.204) or the interaction between land use and landscape (*F*_3,24_ = 0.702, *p* = 0.927, Wilks Lambda 0.702; *w*_3,24_ = 5.665, *p* = 0.100). Both ordination methods recovered three distinct groups: (1) rainforest and jungle rubber, (2) rubber and (3) oil palm (NMDS Fig. 3; LVM Fig. S6). Overall, the majority of salticid spider morphospecies occurred in jungle rubber and rainforest with 26 species present in one or both systems (Fig. S4B).

**Figure 4:**
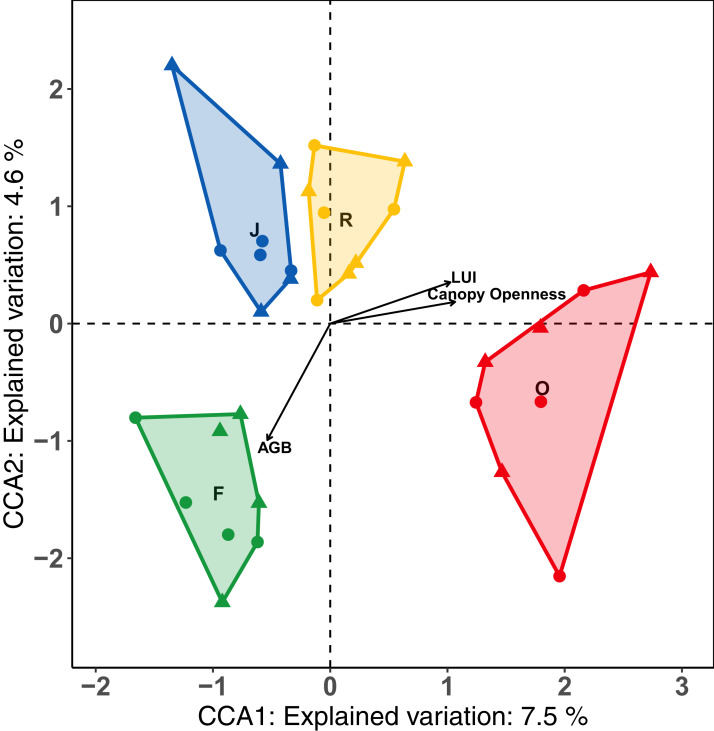
Canonical correspondence analysis (CCA) biplot of relationships between salticid spiders and environmental constraints across four land-use systems. F = rainforest, J = jungle rubber, R = rubber, O = oil palm. Raw data for each plot within the two landscapes are given as circles (Bukit Duabelas) and triangles (Harapan). Environmental variables significantly affecting canopy salticid spiders according to ANOVA are shown as arrows. AGB = aboveground biomass; LUI = land use intensity.

The first axis of the CCA separated the land-use systems along a gradient of land-use intensity and canopy openness, and accounted for 7.5% of the variance (Fig. 4). The second axis separated land-use systems along the gradient of aboveground biomass and explained 4.5 % of the variance. Results of the forward selection procedure indicated that LUI (*F* = 2.39, *p* = 0.001, *R*^2^_a_ = 0.0433 = 4.3%), AGB (*F* = 1.53, *p* = 0.013, *R*^2^_a_ = 0.0157 = 1.57%) and canopy openness (*F* = 1.46, *p* = 0.017, *R*^2^_a_ = 0.0157 = 1.57%) significantly influenced the assemblages of salticid spiders, together explaining 7.4% of the variance. Temperature and SSCI did not significantly improve the model. Oil palm samples, which had the highest LUI and canopy openness of all the land-use systems, clustered on the right half of the CCA. By contrast, rainforest plots, with low LUI, dense canopies and high AGB, were positioned in the lower left of the CCA biplot. Rubber was positioned in the upper centre of the ordination as it has the lowest values for AGB, but lower land-use intensity and canopy openness than oil palm. Jungle rubber was intermediate for all three environmental variables and clustered in the upper left corner of the CCA.

**Figure 5:**
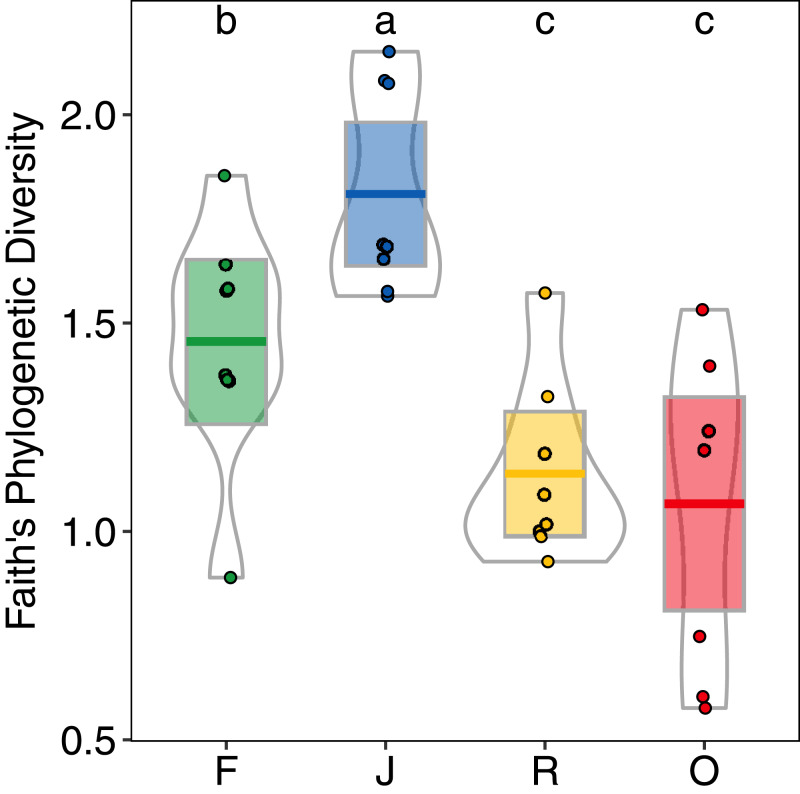
Faith’s Phylogenetic Diversity (PD) of arboreal salticid spider communities across four land-use systems. F = rainforest, J = jungle rubber, R = rubber, O = oil palm. Boxplots show mean (horizontal line), 95% confidence interval (box), density distribution (grey lines) and raw data (dots). Different letters above boxplots mark significant differences as indicated by Holms corrected Tukey HSD, *p* < 0.05.

Generally, PD showed similar patterns as morphological species richness as it differed significantly among land-use systems (*F*_3,28_ = 11.98, *p* < 0.001) (Fig. 5), but not between landscapes (*F*_1,27_ = 2.62, *p* = 0.117), and was significantly higher in jungle rubber (PD_J_ = 1.80 ± 0.24, mean ± s.d.) than in all other land-use systems. However, PD in rainforest (PD_F_ = 1.45 ± 0.28) was significantly higher than in both rubber (PD_R_ = 1.14 ± 0.21) and oil palm (PD_O_ = 1.07 ± 0.37) monocultures.

**Figure 6:**
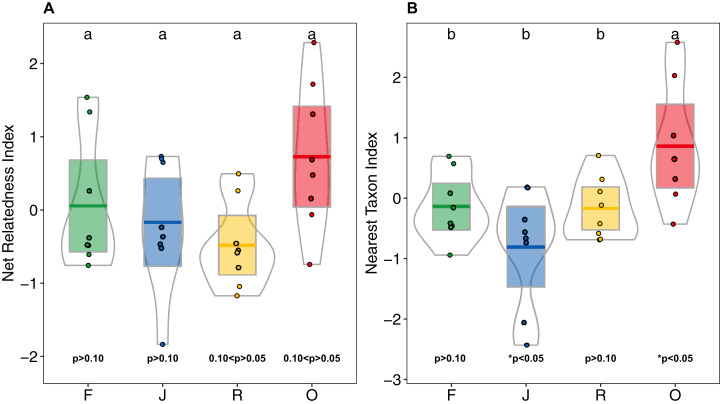
(A) Net Relatedness Index (NRI) and (B) Nearest Taxon Index (NTI) of arboreal salticid spider communities across four land-use systems. F = rainforest, J = jungle rubber, R = rubber, O = oil palm. Boxplots show mean (horizontal line), 95% confidence interval (box), density distribution (grey lines) and raw data (dots). Different letters above boxplots mark significant differences as indicated by Holms corrected Tukey HSD, *p* < 0.05. *P*-values for one sided t-tests against 0 (random assembly) are shown below the boxes. Values of NRI and NTI become positive for phylogenetic clustering, negative for phylogenetic overdispersion and 0 in random assemblies.

Higher NRI and NTI in oil-palm monocultures indicated a trend towards phylogenetic clustering of salticid spider communities, which was not found in the other land-use systems (Figs. 6A and 6B). NTI was significantly affected by land use (anova; *F*_3,28_ = 5.98, *p* = 0.002) but not by landscape (anova; *F*_1,__27_ = 0.481, *p* = 0.493). Comparison to the null model of random assembly revealed phylogenetic clustering of salticid spiders in oil palm (one-sided *t*-test; NTI_O_ = 0.86 ± 1; *t* = 2.43, df = 7, *p* = 0.045) but overdispersal in jungle rubber (NTI_J_ = −0.80 ± 0.95; *t* = −2.38; df = 7, *p* = 0.048); rubber and rainforest showed no significant deviation from random. In pairwise comparisons, NTI was significantly higher in oil palm than in any of the other land-use system. Land-use was not a significant explanatory factor for NRI (*F*_3,27_ = 2.85, *p* = 0.055), nor was landscape (*F*_1,3_ = 0.68, *p* = 0.414). Similar to NTI, NRI values were highest in oil palm (NRI_O_ = 0.72 ± 0.99), but they were not significantly different from those in rubber (NRI_R_ = −0.47 ± 0.58), jungle rubber (NRI_J_ = 0.16 ± 0.86) or rainforest (NRI_F_ = 0.05 ± 0.90). One-sided *t*-tests indicated no significant difference from random assembly for any of the investigated land-use systems (Table S3).

## Discussion

In contrast to our first hypothesis, salticid spider abundance was similar in all four land-use systems studied. This was due to high frequency of several dominant species in monoculture plantations (Fig. S7). Land-use change may increase the abundance of a few generalist spider species (Shochat et al., 2004), resulting in similar or even higher spider abundance in rubber plantations compared to rainforests (Zheng, Li & Yang, 2015; Zheng et al., 2017). Also, contrary to our first hypothesis, species richness was highest in jungle rubber, not rainforest. Jungle rubber is an extensive agroforest system in which rubber trees have been planted into degraded rainforest, and is highly heterogeneous in management practices and plant richness (Rembold et al., 2017; Gouyon, De Foresta & Levang, 1993). At first sight, the observed pattern in salticid spider richness might appear to represent a scenario predicted by the Intermediate Disturbance Hypothesis (IDH; Connell, 1978). IDH predicts maximum species richness at intermediate disturbance, as “too little” disturbance may lead to low diversity through competitive exclusion, while “too much” disturbance may eliminate species incapable of rapid re-colonialization (Hoopes & Harrison, 1998). IDH-like patterns have been described in other East Asian spider communities in response to moderate agricultural management (Tsai, Huang & Tso, 2006; Chen & Tso, 2004). However, in addition to rubber extraction, smallholder farmers in Sumatra use jungle rubber as source of timber and firewood, which over time changes jungle rubber plots from moderately disturbed, rainforest-like ecosystems into ones that resemble rubber monocultures. Given the increasing levels of disturbance in older jungle rubber stands, IDH is a precarious explanation for salticid richness. Also, for a range of other arthropod taxa investigated at the same study sites, average species richness was highest in rainforest, intermediate in jungle rubber and lowest in the monocultures of rubber and oil palm (beetles: Fahri, Atmowidi & Noerdjito, 2016; Kasmiatun et al., 2020; ants: Nazarreta et al., 2020; ground spiders: Potapov et al., 2020; butterflies: Panjaitan et al., 2020).

Interestingly, overall epiphyte richness and variability of epiphyte abundance and richness was highest in jungle rubber as well (Böhnert et al., 2016). Epiphytic plants in trees serve as microhabitats for a range of canopy spiders (Méndez-Castro et al., 2018) and increased epiphyte abundance has been shown to foster the diversity of canopy spider communities (Méndez-Castro & Rao, 2014; Stuntz et al., 2002). Salticid richness may therefore be explained by the more complex and prevalent epiphyte communities in jungle rubber, compared to the other three land-use systems. Overall, similar to the other taxa mentioned above, a substantial proportion of the observed salticid species were exclusively found in rainforest and/or jungle rubber (ca. 40%, that is, 28 of 70 species), while a much smaller proportion were exclusive to rubber and/or oil palm (20%, that is, 15 of 70 species). This highlights the importance of both rainforest and jungle rubber for the conservation of salticid biodiversity, a consideration for conservation policy recommendations.

Forest conversion to monoculture plantations strongly shifted the community composition of salticid spiders, as predicted by our second hypothesis. This pattern is mirrored in other arthropods along the same land-use gradient, including ants (Nazarreta et al., 2020), butterflies (Panjaitan et al., 2020), or beetles, butterfly caterpillars, parasitoid wasps, flies, springtails and true bugs (EFForTS Newsletter, 2018, 2020). The shift in salticid community composition is mediated by environmental factors including canopy openness, aboveground tree biomass and land use intensity. Such indicators of habitat complexity are known determinants for the structure of spider communities, with complex and diverse habitats promoting spider diversity (Floren & Deeleman-Reinhold, 2005; Pinkus-Rendon, Leon-Cortes & Ibarra-Nunez, 2006).

Canopies of oil palm and rubber form monotonous expanses (Zheng, Li & Yang, 2015; Zemp et al., 2019a) compared to the complex canopies of rainforest and jungle rubber comprising different tree species. These structural differences are likely responsible for the fundamentally different composition and species richness of salticid spider communities in monoculture plantations. One important parameter of canopy complexity is canopy openness, which significantly influenced salticid spider assemblages in this study. Canopy cover of rubber and oil palm plantations was significantly lower compared to the dense multi-layer canopies in rainforest and jungle rubber (Drescher et al., 2016). AGB, which increases with tree age and height, was also a significant predictor of community compositions of salticid spiders. Tree age, without consideration of biomass, has previously been recognized as an important factor for canopy spiders in Southeast Asian rainforests (Floren et al., 2011) and epigean spider assemblages in European spruce forests (Purchart et al., 2013). Finally, the land use intensity index (LUI), derived from quantities of fertilizer and herbicides applied, and the number of planted oil palms or rubber trees per hectare (Brinkmann et al., 2019), significantly explained community composition of salticid spiders. It is important to note that this result could be influenced by LUI being set to 0 for rainforests (Brinkmann et al., 2019), potentially exaggerating contrasts among land-use systems.

The overall low explanatory power of our environmental variables suggests the influence of other factors on salticid spider community composition. Epiphytic plants, for example, increase habitat complexity and positively affect spider abundance and diversity, thus influencing spider community composition and ecosystem functioning (Méndez-Castro & Rao, 2014; Méndez-Castro et al., 2018, 2020). Rainforest conversion to oil palm and rubber monocultures entails a shift from abundant, species-rich, angiosperm dominated epiphyte communities in rainforest and jungle rubber, to equally abundant, but species poor, fern-dominated epiphyte communities in oil palm, while epiphytes in rubber are rare and least diverse (Böhnert et al., 2016). Furthermore, epiphytes in oil palm support lower overall arthropod abundance and biomass than epiphytes in rainforest (Turner & Foster, 2009). It is thus reasonable to assume that epiphytes substantially influence canopy salticid communities, but other, currently unknown, factors likely also play a role. Whatever those factors may be, our results support the well-studied association of structural complexity with spider diversity and its impact on spider community composition (Floren & Deeleman-Reinhold, 2005; Halaj, Ross & Moldenke, 2000; Stenchly et al., 2011; Pinkus-Rendon, Leon-Cortes & Ibarra-Nunez, 2006; Zheng, Li & Yang, 2015; Ziesche & Roth, 2008).

Generally, cash-crop plantations of rubber and oil palm are intensively managed monocultures, but there are significant differences in management practices between large-scale estates and various forms of smallholder cash-crop cultivation (see Dislich et al., 2017; Bessou et al., 2017; Formaglio et al., 2020). The management practices currently in place aim to increase yield or gross margin, typically at the expense of biodiversity (Clough et al., 2016; Drescher et al., 2016; Grass et al., 2020). There is increasing evidence, however, that reduced management intensity in oil palm plantations benefits spider communities (Spear et al., 2018), but does not reduce yield (Prescott, Edwards & Foster, 2015) and may even increase economic performance (Darras et al., 2019). Similarly, experimental “biodiversity enrichment” (Teuscher et al., 2016) suggests that planting islands of mixed tree species into an oil palm monoculture can raise levels of biodiversity and ecosystem functioning (Zemp et al., 2019a, 2019b), while increasing oil palm yield (Gérard et al., 2017).

### Phylogenetic patterns

The decline of PD in monoculture plantations supports our third hypothesis, as low phylogenetic diversity in animal and plant communities often coincides with low phenotypic diversity, which is in turn associated with high redundancy in the functional traits that contribute to ecosystem functioning (Díaz et al., 2013; Grab et al., 2019; Turley & Brudvig, 2016). Along this rainforest conversion gradient, low PD of salticids in rubber and oil-palm monocultures implies a limited ability of these communities to support ecosystem functioning, for example, by mitigating pest outbreaks. This likely extends to spider communities of several families, not just salticids, as overall spider richness is lower in rubber and oil palm compared to rainforest and jungle rubber (Potapov et al., 2020; Zheng, Li & Yang, 2015; Zheng et al., 2017). Low diversity may reduce the range of functional traits (e.g., body size and hunting strategy) in a community, which determines the prey spectrum and functional effects of spiders on prey (Cardoso et al., 2011; Schuldt et al., 2014), and ultimately weaken pest suppression by the loss of feeding niches (Kruess & Tscharntke, 1994). Abundance also plays a role in the ability of a species or community to exert predatory control over another species or community (Griffiths et al., 2008), so it might be assumed that similar average salticid abundances across land uses reflect similar biological control capabilities in rubber and oil palm as in rainforest and jungle rubber. However, species-poor predator communities with high functional trait overlap are universally outperformed in ecosystem services, such as biological control, by multi-species predator communities with high levels of functional complementarity (Birkhofer et al., 2008; Griffiths et al., 2008; Kruess & Tscharntke, 1994; Srivastava et al., 2012). Thus, high PD in rainforest and particularly jungle rubber indicates more robust ecosystem functioning.

The establishment of rubber amidst rainforest trees, which is associated with greater epiphyte diversity (Böhnert et al., 2016), may have created new niche space, allowing salticid communities in jungle rubber to exceed the PD of rainforest communities. This could suggest jungle rubber as a sustainable alternative to monoculture plantations of rubber and oil palm, as it is inhabited by a highly diverse salticid spider community and simultaneously provides smallholder farmers with income through the sale of rubber. However, jungle rubber is disproportionately less lucrative and more labour intensive than monoculture plantations (Clough et al., 2016; Grass et al., 2020). In the absence of monetary or other incentives, for example, by governmental bodies, jungle rubber increasingly exists only as a transitional step when a lack of investment capital has hampered the transformation into more lucrative monoculture systems and does not ultimately conserve or foster biodiversity.

Phylogenetically clustered communities in oil palm, as predicted by our third hypothesis, suggest environmental filtering as the predominant process in structuring salticid spider communities in these monoculture plantations. Presumably, harsh and frequently changing environmental conditions in monoculture plantations function as filter favouring colonization by closely related, likely generalist, species (Srivastava et al., 2012). In Contrast to oil palm plantations, NTI of salticid spider communities in jungle rubber suggests phylogenetic overdispersion and therefore competition as the main factor influencing community assembly. This contradicts the assumption that disturbance reduces interspecific competition and further argues against the intermediate disturbance hypothesis (Connell, 1978; Catford et al., 2012) as an explanation for the high species richness in jungle rubber. In contrast to jungle rubber and oil palm, NTI suggests random assembly of salticid spider communities in rainforest and rubber. The NRI results did suggest phylogenetic clustering in oil palm plantations, but generally lacked statistical power to detect non-random patterns. The difficulty in drawing consistent conclusions on community assembly patterns based on NRI and NTI has been stressed previously (Jarvis et al., 2017; Muscarella et al., 2014; Vamosi et al., 2009) and the categorical nature of their underlying assumptions has been criticized (Narwani et al., 2015). Indeed, the interpretation that phylogenetic and phenotypic clustering infers environmental filtering while competition is represented by phylogenetic and phenotypic (over) dispersion may be unwarranted, as habitat filtering and competition can interact with each other by additive or opposing effects (Gerhold et al., 2015; Mayfield & Levine, 2010). As phylogenetic evenness despite low phylogenetic diversity in rubber monoculture plantations and the low percentage of variability explained in the CCA indicate, community assembly is influenced by difficult to identify interacting factors and stochastic effects (Farnon Ellwood, Manica & Foster, 2009; Lepori & Malmqvist, 2009).

## Conclusion

This study is the first comprehensive assessment of the impacts of rainforest transformation to rubber and oil palm plantations on the salticid spider fauna. Using a combination of morphological and molecular sequence data, we showed that salticid spider communities in monoculture plantations differ fundamentally from those in less intensively managed systems, that is, rainforest and jungle rubber. We found lower species richness in rubber plantations and substantially less phylogenetic diversity in communities of both rubber and oil palm plantations than in rainforest and jungle rubber. Habitat filtering was identified as the major structuring force in salticid spider community assembly in oil palm plantations. Aboveground plant biomass, canopy openness and land-use intensity are important predictors of salticid spider community structure. Our data emphasises the importance of rainforest and jungle rubber for the conservation of canopy salticid spiders and the ecosystem services they contribute to, a pattern observed in a range of animal and plant taxa from the study region. The combined analysis of morphological and molecular data as adopted in the present study is a promising approach for analysing the response of other taxa to the conversion of rainforest into plantation systems and presents a starting point for understanding the structuring forces of the largely undescribed spider communities of tropical ecosystems.

## Supplemental Information

10.7717/peerj.11012/supp-1 Supplemental Information 1 Location of the 32 study plots in Jambi Province, Sumatra, Indonesia, arranged in two landscapes near Bukit Duabelas National Park and Harapan Rainforest. Land-use systems are coded by color (green = old growth secondary lowland rainforest, blue = jungle rubber, yellow = rubber, red = oil palm).

10.7717/peerj.11012/supp-2 Supplemental Information 2 Pearson correlation coefficients between all pairs of environmental variables used in the canonical correspondence analysis given as values and scatter plots.

10.7717/peerj.11012/supp-3 Supplemental Information 3 Bayesian inference phylogeny for the 28S rDNA alignment of 55 species of salticid spiders. Morphospecies numbers are aligned to tip labels and Bayesian posterior probabilities are given for each node.

10.7717/peerj.11012/supp-4 Supplemental Information 4 Venn diagrams showing the number of arboreal salticid spider morphospecies. (A) Number of morphospecies in the four land-use systems forest (green), jungle rubber (blue), rubber (yellow) and oil palm (red). (B) Number of morphospecies in the two landscapes Bukit Duabelas (yellow) and Harapan (purple).

10.7717/peerj.11012/supp-5 Supplemental Information 5 Species accumulation curves of arboreal salticid spider communties across four land-use systems. Green = rainforest, blue = jungle rubber, yellow = rubber, red = oil palm. Species accumulation curves were calculated with 999 permutations and sites were added in random order.

10.7717/peerj.11012/supp-6 Supplemental Information 6 Pure latent variable ordination of arboreal salticid spider communities across four land-use systems. Salticid species (+) in plots (circles= Bukit Duabelas, triangles = Harapan) of rainforest (green), jungle rubber (blue), rubber (yellow) and oil palm (red) shown as latent variable means with a negative binomial distribution. Ellipses represent 75% confidence intervals.

10.7717/peerj.11012/supp-7 Supplemental Information 7 Rank abundance curves of arboreal jumping spiders. Shape of the rank abundance curves differed significantly between land-use systems under ANOVA (*F*_3_ = 17.21, *p* < 0.001). Best model fits as per Akaike Information Criterion (AIC): Mandelbrot for rainforest (green) and jungle rubber (blue). Preemption for rubber (yellow) and Zipf-model for oil-palm (red).

10.7717/peerj.11012/supp-8 Supplemental Information 8 GenBank accession numbers of salticid spider sequences and Basic Local Alignment Search Tool (BLAST) hits of highest percent identity. Column [1]:Genera derived from morphology-based identification. Column [2,3]: Genbank Accession numbers for acquired *COI* and 28S rDNA sequences of salticid spiders. Column [4]: Genus according to BLAST results with highest similarity. Column [5,6]: Morphospecies and their associated unique Identifier. Column [7,8,9,10]:*COI* fragment lenghts and BLAST-hits of similarity. Column [11,12,13,14]: 28S rDNA fragment lenghts and BLAST-hits of highest similarity. Cells with ’---’ mark morphospecies without acquired sequences.

10.7717/peerj.11012/supp-9 Supplemental Information 9 Grouping results of ABGD for *COI* and 28S rDNA with merged morphospecies and the resulting set 55 of unique taxa used in the calculation of the Bayesian inference tree for 28S rDNA. Eight morphospecies were grouped into four unique molecular taxa by the inferred barcode gap for 28S rDNA and *COI* (yellow). New candidate species after ABGD grouping results (024, 027, 056, 086) are underlined in the third column.

10.7717/peerj.11012/supp-10 Supplemental Information 10 T-test results for NRI and NTI in each land-use system.

10.7717/peerj.11012/supp-11 Supplemental Information 11 Raw sampling and environmental data.

10.7717/peerj.11012/supp-12 Supplemental Information 12 Sequence alignments and batch file used for the analysis.

10.7717/peerj.11012/supp-13 Supplemental Information 13 R-code used for the analysis.

## Additional Information and Declarations

### Field Study Permissions

The following information was supplied relating to field study approvals (i.e., approving body and any reference numbers): This study was conducted using canopy spiders sampled based on Research Permits 204/SIP/FRP/SM/VI/2012, 27/EXT/SIP/FRP/SM/IV/2013 and 131/SIP/FRP/E5/Dit.KI/V/2017 (Ministry of Research and Technology/National Research and Innovation Agency, Indonesia, RISTEKDIKTI), Collection Permit S.710/KKH-2/2013 (The Directorate General of Forest Protection and Nature Conservation, Indonesia, PHKA) and Export Permit SK.61/KSDAE/SET/KSA.2/3/2019 (by the The Directorate General of Nature Resources and Ecosystem Conservation, Indonesia, KSDAE). Access to individual field sites (plots) in privately owned farmland was granted by 35 individual smallholders in individual agreements between them and University of Jambi UNJA, the EFForTS partner University in Jambi Province, Indonesia. Access to field sites in Harapan Rainforest was granted through a bilateral agreement between UNJA and PT REKI (Perseroan Terbatas Restorasi Ekosistem Indonesia) which manages Harapan Rainforest. Access to field sites in Bukit Duabelas National Park was similarly granted through a bilateral agreement between UNJA and the National Park administration. All related agreements and contracts between UNJA and stakeholders can be provided upon request (in Indonesian only).

### DNA Deposition

The following information was supplied regarding the deposition of DNA sequences: The 28S rDNA Sequences are available at GenBank: MT596625–MT596683. The COI Sequences are also available at Genbank: MT671511–MT671557 and MT672319–MT672335.

### Data Availability

The following information was supplied regarding data availability: Raw data and R code are available in the Supplemental Files.
