# Supplementary figures and images for: Changes in diversity and community assembly of jumping spiders (Araneae: Salticidae) after rainforest conversion to rubber and oil palm plantations
Source: PeerJ. 2021 Mar 4;9:e11012. doi: 10.7717/peerj.11012 (PMC7937343; doi:10.7717/peerj.11012)

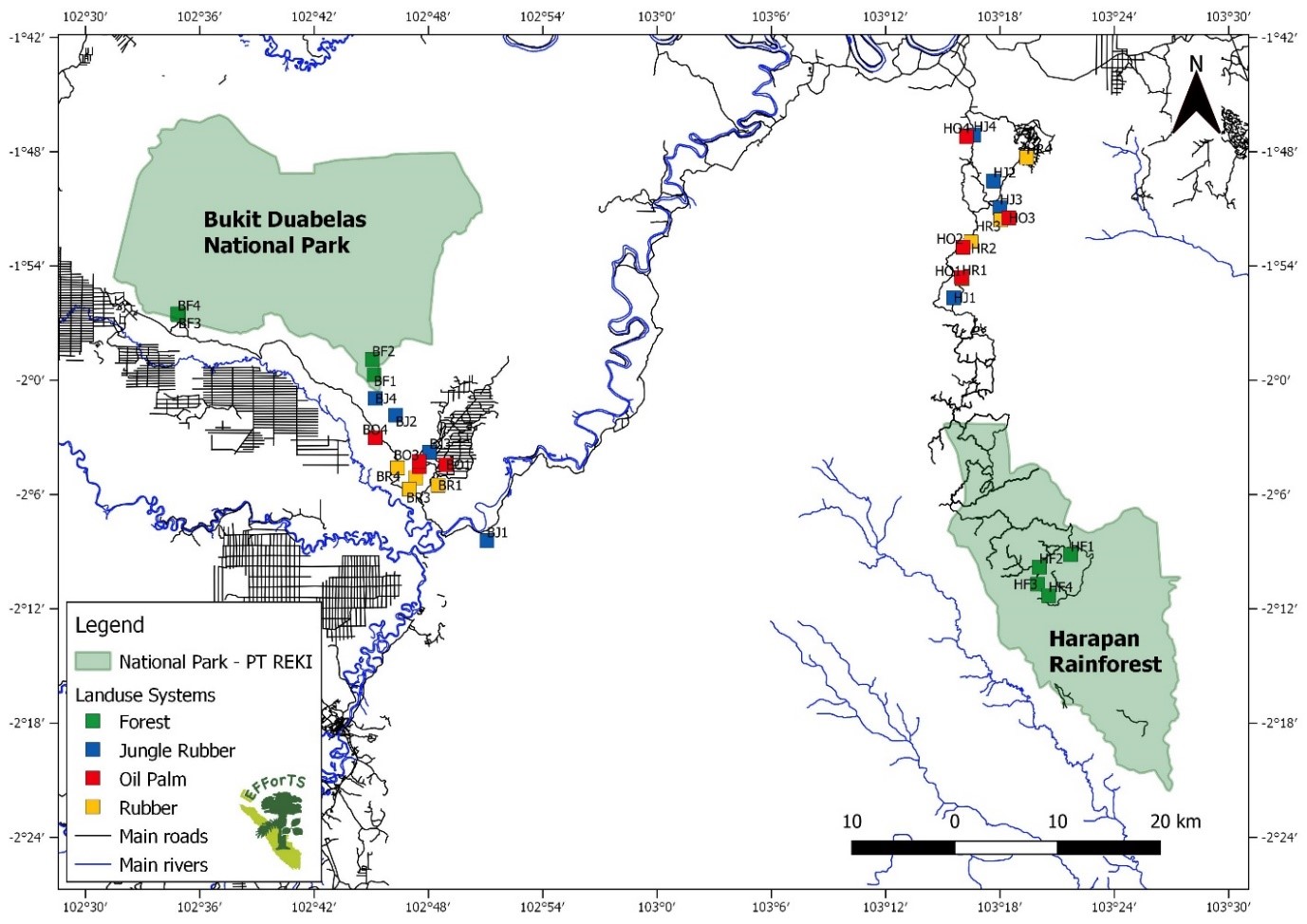

Supplement: Supplemental Information 1 — Land-use systems are coded by color (green = old growth secondary lowland rainforest, blue = jungle rubber, yellow = rubber, red = oil palm). [file peerj-09-11012-s001.jpg]

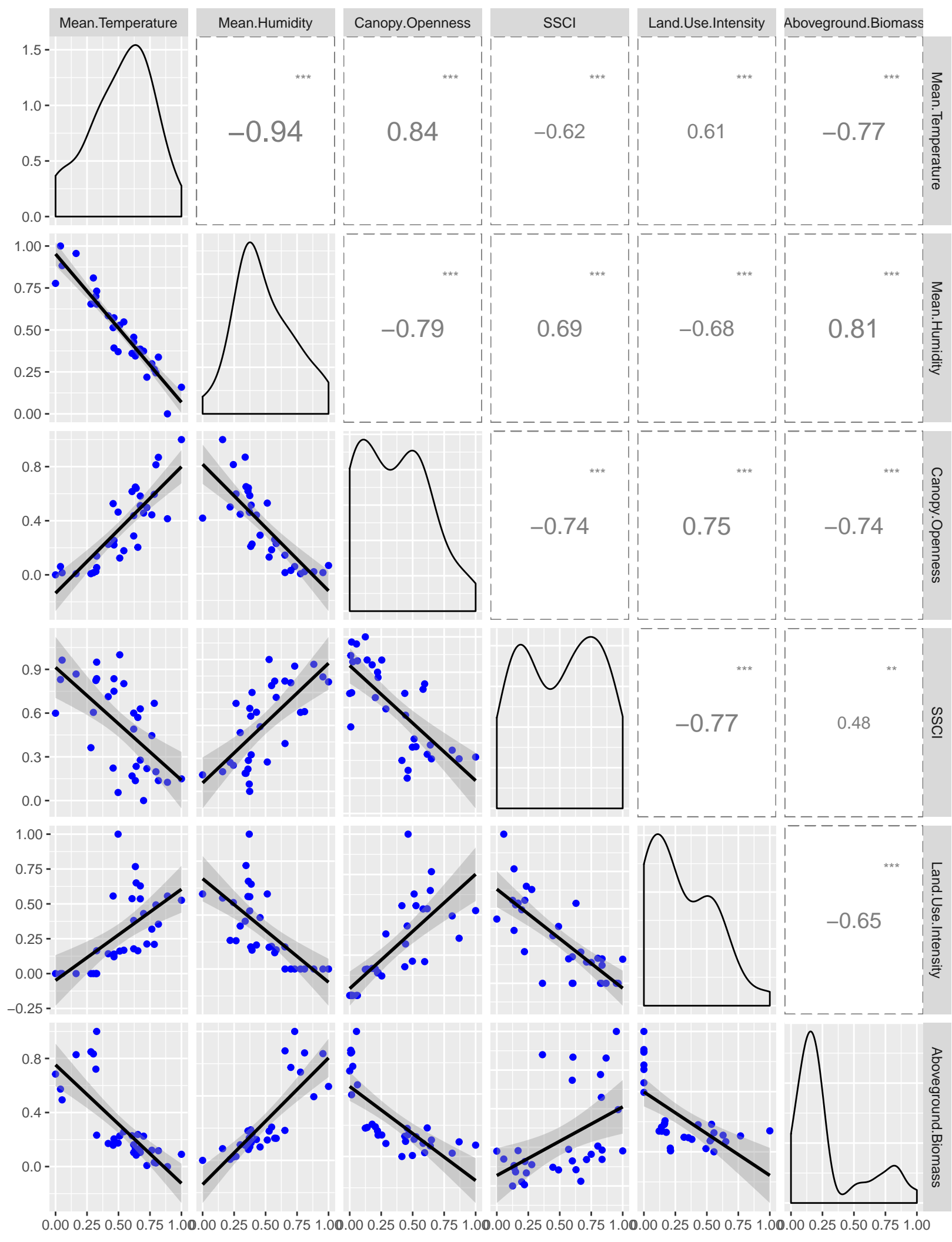

Supplement: Supplemental Information 2 [file peerj-09-11012-s002.pdf]

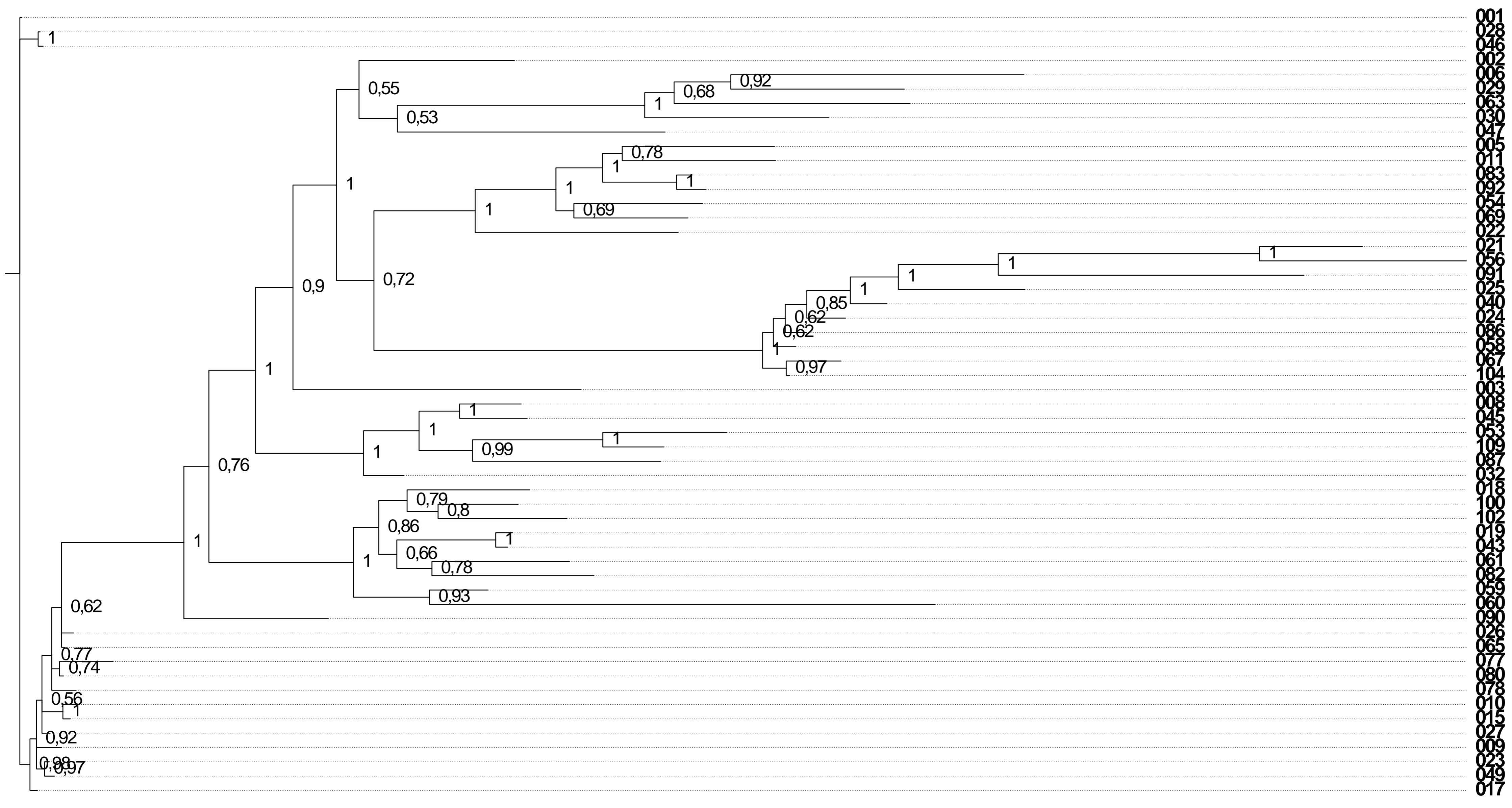

Supplement: Supplemental Information 3 — Morphospecies numbers are aligned to tip labels and Bayesian posterior probabilities are given for each node. [file peerj-09-11012-s003.pdf]

**a**

Bukit Duabelas

Harapan

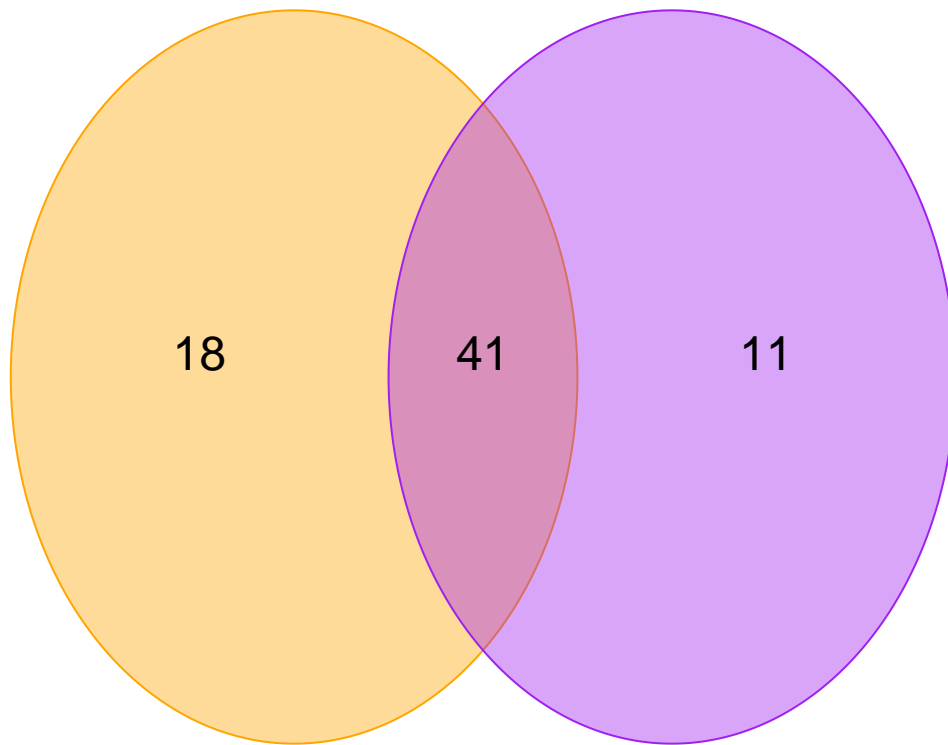

**b**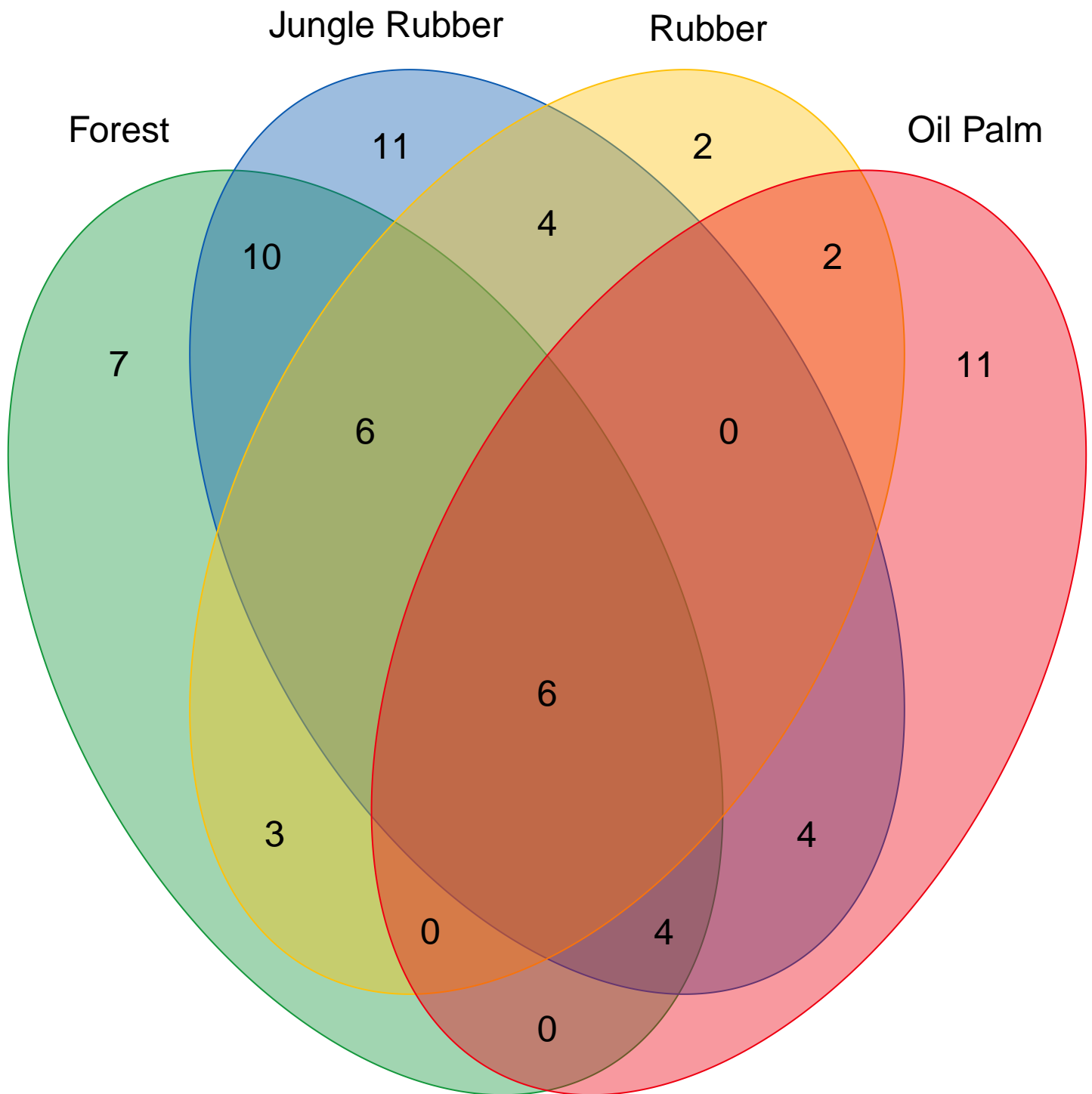

Supplement: Supplemental Information 4 — (A) Number of morphospecies in the four land-use systems forest (green), jungle rubber (blue), rubber (yellow) and oil palm (red). (B) Number of morphospecies in the two landscapes Bukit Duabelas (yellow) and Harapan (purple). [file peerj-09-11012-s004.pdf]

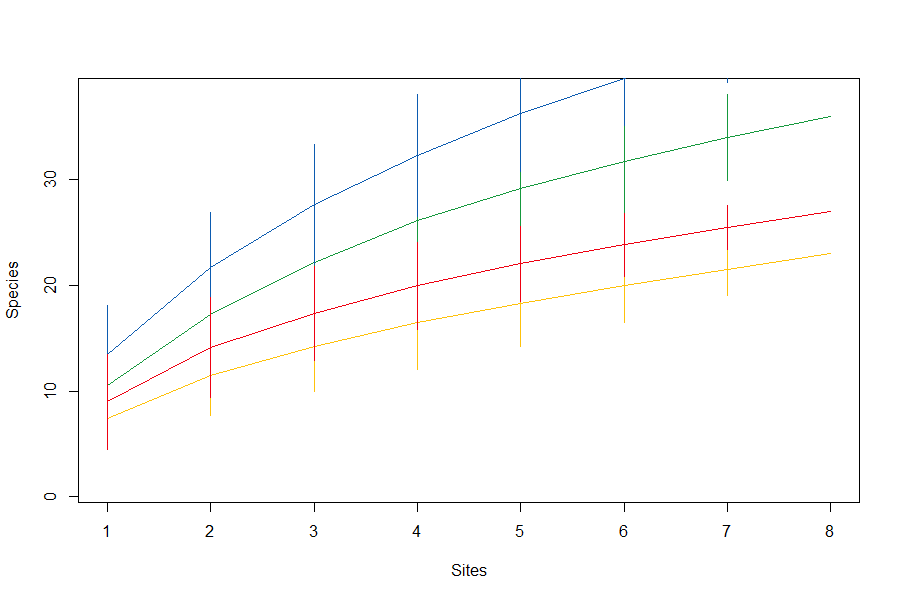

Supplement: Supplemental Information 5 — Green = rainforest, blue = jungle rubber, yellow = rubber, red = oil palm. Species accumulation curves were calculated with 999 permutations and sites were added in random order. [file peerj-09-11012-s005.png]

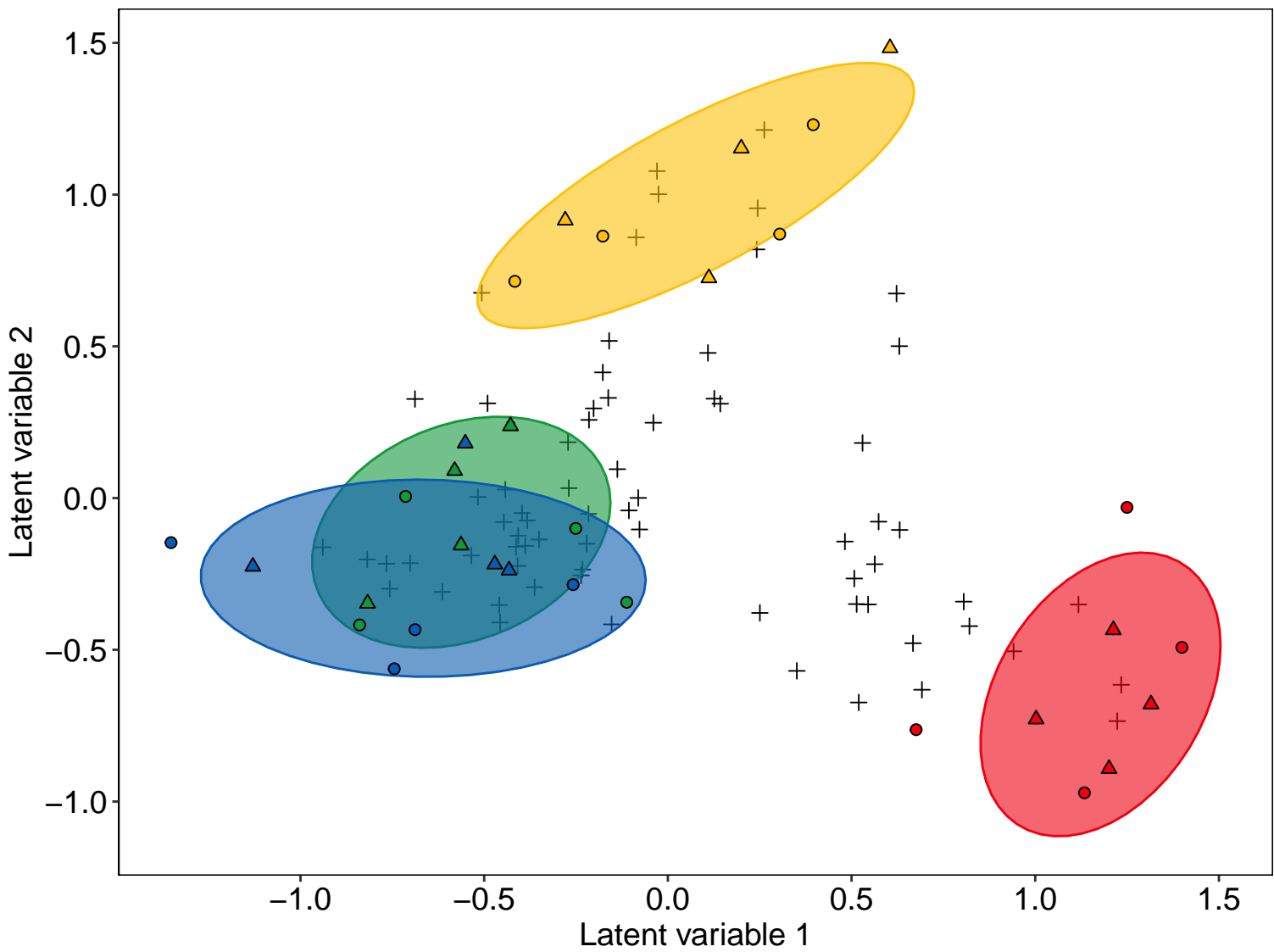

Supplement: Supplemental Information 6 — Salticid species (+) in plots (circles= Bukit Duabelas, triangles = Harapan) of rainforest (green), jungle rubber (blue), rubber (yellow) and oil palm (red) shown as latent variable means with a negative binomial distribution. Ellipses represent 75% confidence intervals. [file peerj-09-11012-s006.pdf]

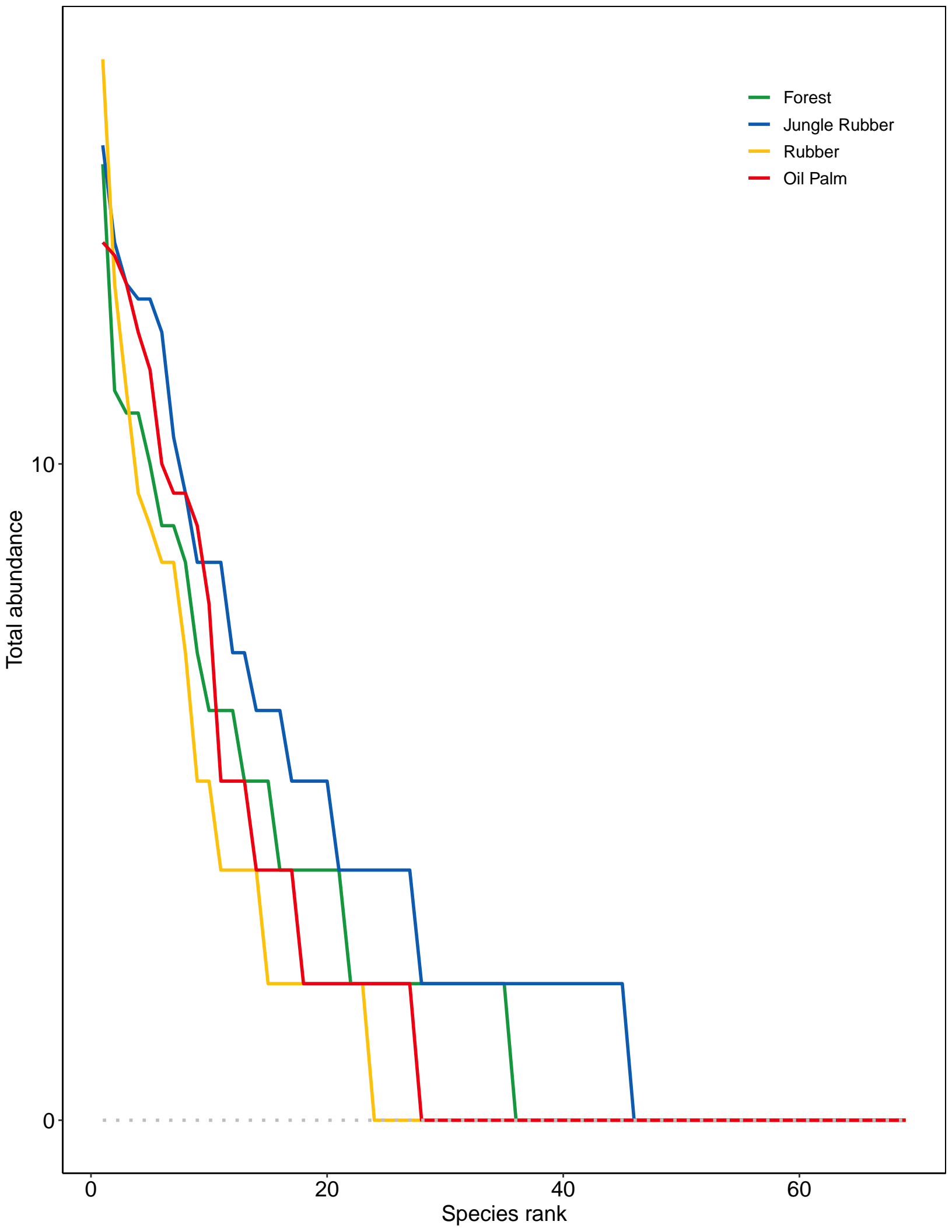

Supplement: Supplemental Information 7 — Shape of the rank abundance curves differed significantly between land-use systems under ANOVA (F3 = 17.21, p < 0.001). Best model fits as per Akaike Information Criterion (AIC): Mandelbrot for rainforest (green) and jungle rubber (blue). Preemption for rubber (yellow) and Zipf-model for oil-palm (red). [file peerj-09-11012-s007.pdf]
